# Profiling of tumour board characteristics and functioning for cancer care under hospitals registered under National Cancer Registry Programme in India

**DOI:** 10.3332/ecancer.2026.2090

**Published:** 2026-03-12

**Authors:** Gokul Sarveswaran, Jayasankar Sankarapillai, Kondalli Lakshminarayana Sudarshan, Prashant Mathur

**Affiliations:** ICMR National Centre for Disease Informatics and Research, Bengaluru, Karnataka 562110, India

**Keywords:** cancer care systems, cancer care quality, national cancer registry programme, oncology practices, tumour board functioning

## Abstract

**Background:**

Multidisciplinary tumour board (MTB) plays an important role in cancer care by collaborating specialist from various disciplines to discuss diagnosis, treatment plan and patient management. Despite its importance, a systematic evaluations of tumour board in hospital registered under hospital-based cancer registries (HBCRs) is limited. This study aims to assess the characteristics, composition and functioning of tumour board in these hospital to identify gaps and challenges to improve the tumour board operations.

**Methods:**

The study was conducted from October 1st to 31st 2024 using cross sectional design among hospital under HBCR. A set of structured questionnaires was administered using Google Form, which consists of both qualitative and quantitative questionnaires. Descriptive statistics and chi-square test were used to analyze the quantitative data, while manual thematic analysis was carried out to analyze the qualitative responses.

**Result:**

Of 229 hospitals, only 172 (75%) responded. Among them, 137 (79.7%) reported having a functional tumour board. Most of the tumour boards were established in tertiary care centres (45.3%) and medical colleges (43.6%). In-person meeting (73.7%) was the most common with 24.8% following a hybrid model. Only 5.1% of hospitals engaged in cross-hospital case discussions; 16.8% maintained electronic medical record notes; and 48.2% lacked any follow-up mechanisms to track tumour board recommendations and outcomes. Involvement of palliative care specialists and other supportive paramedical health professionals was low. In addition to that, thematic analysis also identified that there was inadequate documentation, inconsistent follow-up and limited tumour board participation.

**Conclusion:**

Although MTBs are established widely in most of the HBCR-affiliated hospitals, variation exist in structure, composition and functionality. Strengthening MTBs through standardised protocol, participation of diverse specialities, increased cross-hospital collaboration, virtual tumour boards and proper documentation practices through electronic data recording systems could enhance the decision making and cancer care outcomes.

## Introduction

Cancer is one of the leading causes of death across the world and in India. Despite the economic development and advancement in medical technologies, there is a rising incidence of cancer globally and in India [1]. This is majorly attributed to an increase in behavioural and environmental risk factors related to cancer. Cancer management requires adequate knowledge and clinical skills from diverse specialties to deliver a high standard of quality cancer care. Hence, the Multidisciplinary tumour boards (MTBs) play a significant role in cancer management due to their complex nature. MTB functions by an approach to treatment planning through the involvement of health care professionals from different specialities. These professionals focus on reviewing and discussion of cancer stage, histology type and other pathological/molecular characteristics of tumour and recommend the best treatment plans having better patient outcomes [2].

MTB are conducted worldwide for cancer management. Several studies reported that there are substantial alterations in diagnosis and treatment strategies, which authenticated the MTB for decisions making. A multidisciplinary approach is the best way to deliver the complex care needed for cancer management. However, there are organisational and cultural challenges associated with it, which must be led by skilled health managers who can ensure collaboration and promote team work within an organisation [3].

A study done by Saha *et al* [4] in Eastern India reported that involvement of MTB resulted in improved treatment compliance rates by 50% and 52.8% survived after 1 year. Other available studies in the literature showed that cancer patients with a history of three or more MTB discussion showed a significantly better overall survival than patients with no MTB discussions. The aim of MTB is to utilise the collective knowledge from group specialists to provide a higher quality of institutional care [5].

The National Cancer Registry Programme (NCRP) is a national initiative that aimed at collecting and analysing the data related to cancer, providing reliable statistics on incidence, distribution and trends of cancer for India. NCRP operates through networks of Population-based cancer registries (PBCRs) and hospital-based cancer registries (HBCRs). PBCR collects data on cancer incidence and mortality from a defined geographic population. PBCR provides statistics on cancer incidence, mortality, trends and effectiveness of cancer prevention and control programs within the defined population. Contrarily, HBCR gathers data from hospitals and health care facilities that diagnose and treat cancer. The data from HBCR will include detailed information on cancer diagnosis, stage, type of treatment provided and treatment outcomes. This will help in understanding the cancer presentation patterns at the treatment centres, stage and treatment selection in clinical practice that are crucial for improving cancer care and treatment protocols.

Despite the recognition of the importance of MTB, hospitals registered under HBCR are not being evaluated systematically. So as to strengthen them, this study aims to address the gap by conducting a survey-based assessment of tumour boards in hospitals registered under HBCR network. There is a great need to evaluate the structure, processes and outcomes associated with tumour boards and provide information about effectiveness, need of improvement in cancer care practices. To improve the tumour board functionality, qualitative insights from health care professionals have also been collected.

## Methods

This cross-sectional study on tumour boards of all hospitals registered as HBCRs under NCRP network was carried out during October 1st to 31st, 2024. Information was collected through a structured survey via Google Form, which included comprehensive details regarding the structure, functioning and clinical effects of the MTB.

The questionnaire contained sections on hospital information, details of principal investigator (PI) for HBCR, the existence and categories of tumour boards and meeting formats (in-person, online or hybrid). Questions on MTB governance were the existence of a secretariat and the composition of the multidisciplinary team. MTB operational questions included the frequency of meetings, criteria for case presentations and processes for decision-making. Additional information on MTB recommendations, documentation practices, follow-up mechanisms for adherence to MTB recommendations, patient outcomes, loss to follow-up, along with audit frequency of tumour board functioning.

Along with the quantitative survey, a qualitative exploration was carried out using open-ended questions with healthcare professionals involved in tumour board operations on challenges and methods to improve MTB functionality. The objective was to have a comprehensive understanding of tumour board functions, including organisational structure, staffing issues and collaboration between institutions.

The respondents for the survey were majorly HBCR PI/co-Principal Investigator (69.8%), followed by Nodal person responsible for tumour board operations at respective hospitals (28.5%), Chief Administrative Officer/Medical Officer (1.7%).

### Statistical analysis

All categorical variables like presence of MTB, frequency of MTB meetings, case presentation, discussion criteria and so on, were summarised as frequency with proportions. To assess the difference in the presence of TB between public and private hospitals, chi-square test was used. *p* value <0.05 was considered as statistical significant. Thematic analysis was conducted to identify patterns, challenges and best practices related to tumour board implementation. Two researchers independently coded the qualitative responses and themes were refined through consensus to ensure consistency and reliability.

## Results

### General characteristics, establishment and composition of tumour boards

Of the 229 hospitals registered under the NCRP network, 172 responded for the survey with a response rate of 75.1%. Of 172 respondents, 137 (79.7%) reported having some form tumour boards and 35 (20.3%) did not have any tumour board.

Among the 172 hospitals, 52.3% (*n* = 90) were public funded institutions and 47.7% (*n* = 82) were private funded institutions. Tertiary-level centres constituted the largest group (45.3%, *n* = 78), followed by medical colleges (43.6%, *n* = 75). Regional Cancer Centres represented 9.8% (*n* = 17) and 2 (1.2%) were secondary care hospitals. MTBs were most commonly established in tertiary care centres and medical colleges.

**Supplementary Figure 1.**
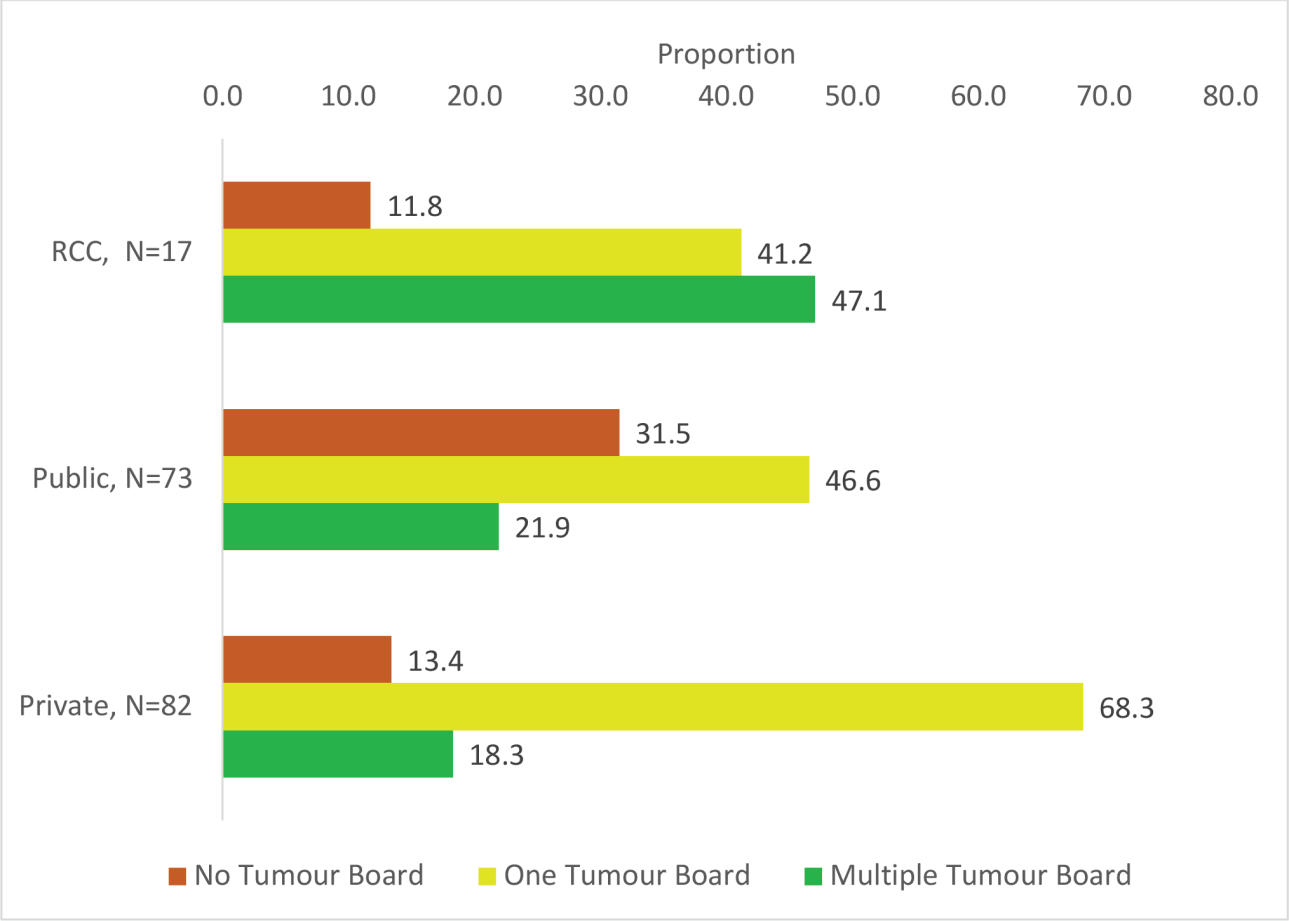
Proportion of functional tumour board at the surveyed hospitals, *N* = 172.

The distribution of the proportion of hospitals with and without tumour board by type of health facility is provided in Supplementary Figure 1.

Of the 137 hospitals that reported having some form of tumour board, 71.5% (*n* = 98) of hospitals operated a single board, while 28.5% (*n* = 39) had multiple boards. Most Tumour Boards (73.7%, *n* = 101) conducted meetings in person, 24.8% (*n* = 34) used a hybrid model (in-person and virtual) and only 1.5% (*n* = 2) conducted fully virtual meetings. A minority of Tumour Boards (5.1%, *n* = 7) participated in regular cross-hospital case reviews and 52.5% (*n* = 72) had a designated secretariat to support their operations (Table 1).

**Supplementary Figure 2.**
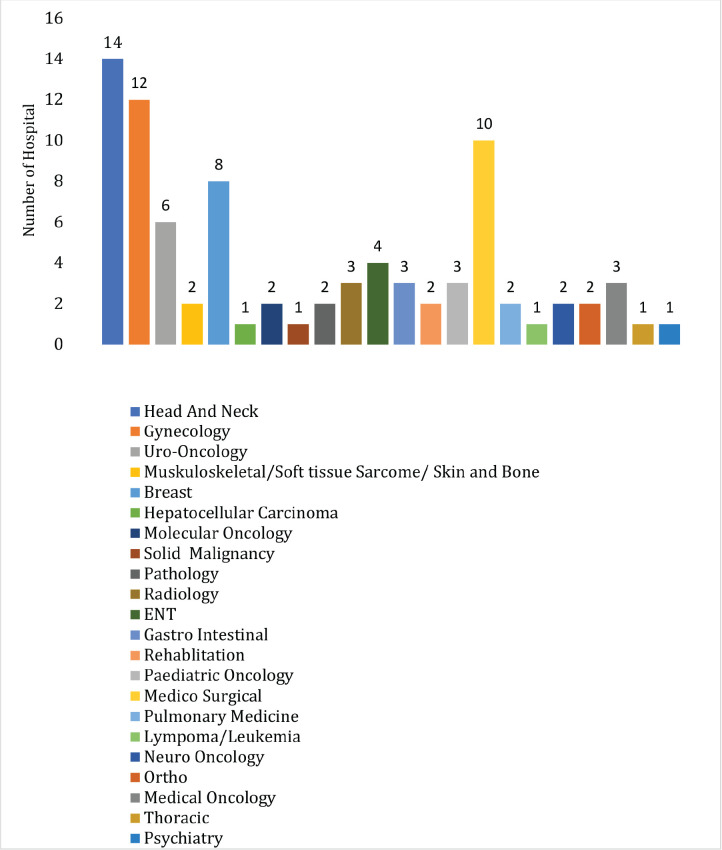
Multiple tumour board by speciality (*N* = 39).

Among hospitals with multiple tumour boards, the most common were those for head and neck, gynaecology, breast, medico-surgical and uro-oncology cancers. Supplementary Figure 2 illustrates the distribution of multiple tumour board across these hospitals.

### Operational procedures, decision-making and outcome tracking

Table 2 describes the operational procedures of tumour board. The frequency of Tumour Board meetings varied across hospitals: 47.4% (*n* = 65) met weekly, 16.8% (*n* = 23) bi-weekly, 2.9% (*n* = 4) monthly and 2.9% (*n* = 4) daily. Notably, 19.0% (*n* = 26) met on an as-needed basis, while others followed irregular schedules (9.5%, *n* = 13). An annual calendar for meetings was maintained by 63.5% (*n* = 87) of tumour boards.

Approximately 52.5% (*n* = 72) of tumour boards focused on specific cancer cases, while 47.5% (*n* = 65) reviewed all cases treated in their respective hospitals.

**Figure 1.**
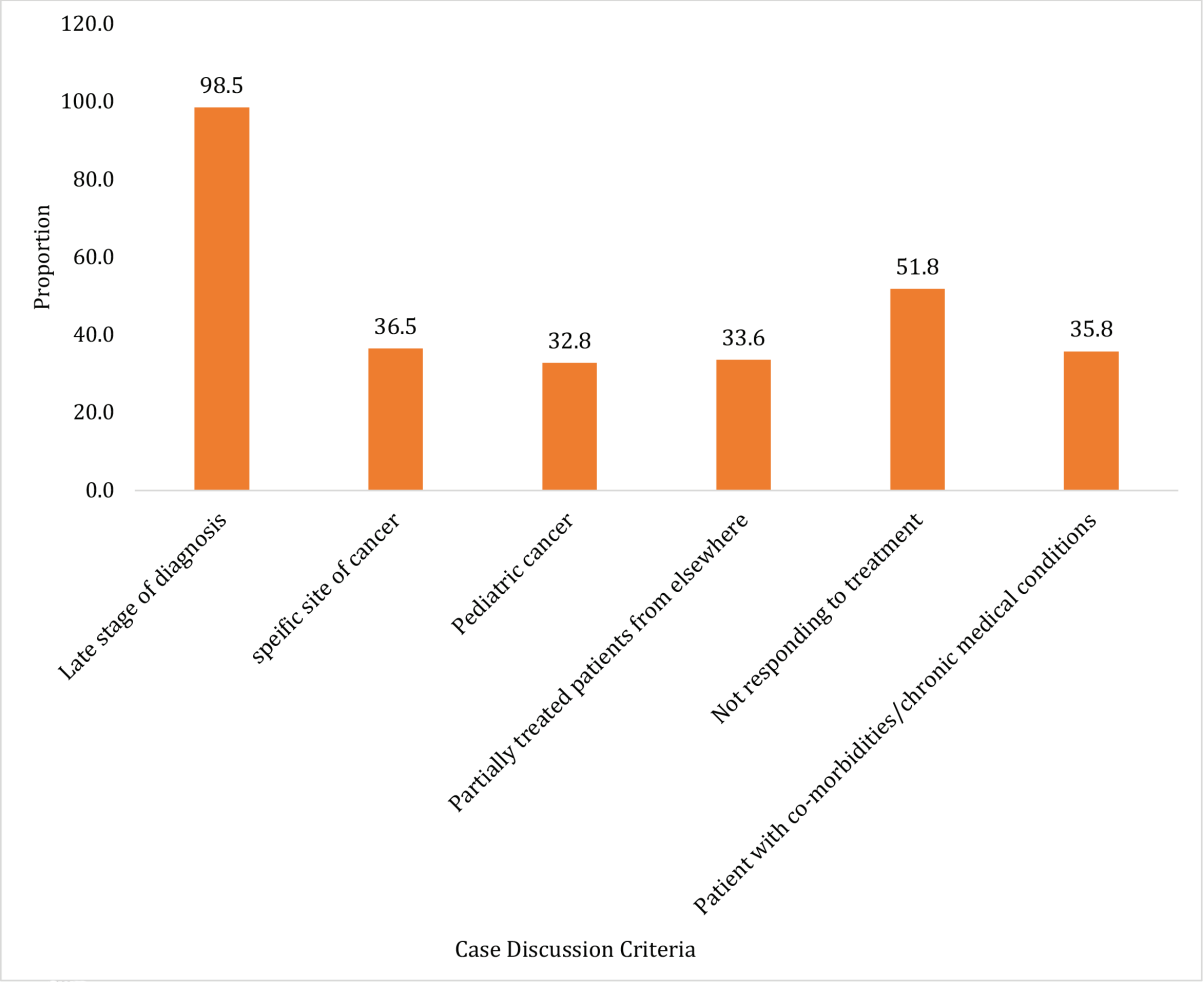
Criteria for presenting special cancer cases in tumour boards, N = 72.

Figure 1 highlights the criteria for presenting special cancer cases in tumour board, with the late stage of diagnosis (98.5%) being the most common. Other significant factor includes treatment resistance (51.8%), specific cancer sites (36.5%), co-morbidities (35.8%), paediatric cancer (32.9%) and partially treated cases (33.6%). These criteria reflects the tumour board focus on complex cases in the meeting.

Table 3 displays the functioning and Outcome Tracking of Tumour Boards Meetings. Documentation methods predominantly involved physical records (63.5%, *n* = 87), with 24.8% (*n* = 34) using a combination of physical and electronic records. Most tumour boards communicated their recommendations through written summaries (38.0%, *n* = 52) or direct interaction with referring physicians (35.0%, *n* = 48). However, only 51.8% (*n* = 71) implemented a follow-up system to monitor the outcomes of recommended treatments.

The implementation rate of tumour board recommendations was reported as ‘most of the time’ by 59.1% (*n* = 81) of respondents and ‘always’ by 29.2% (*n* = 40), reflecting substantial adherence to decisions made during these meetings.

**Supplementary Figure 3.**
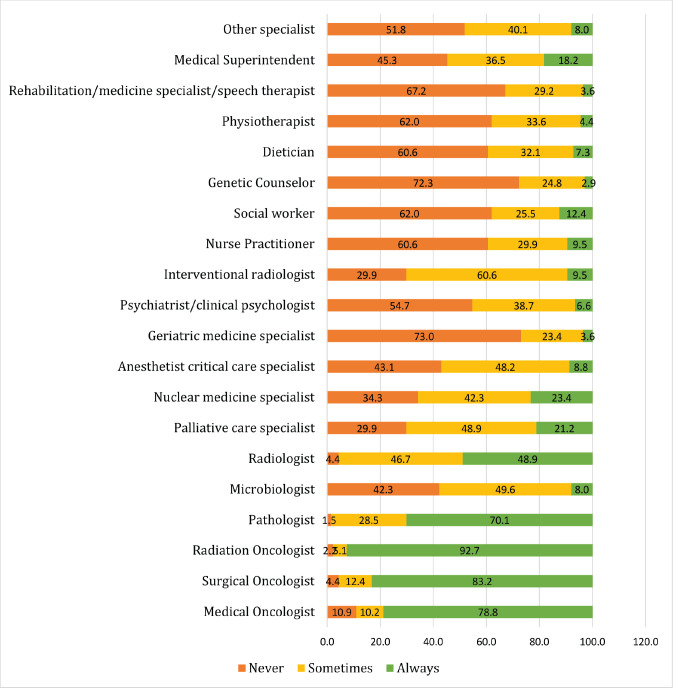
Professional involved in tumour board discussion.

Supplementary Figure 3 reflects the number of professionals who typically took part in tumour board meetings. The three most highly participating specialists were Radiation Oncologists (92.7%), Surgical Oncologists (83.2%) and Medical Oncologists (78.8%). Other specialists, including Pathologists (28.5%), Radiologists (46.7%) and Palliative Care Specialists (48.9%) rarely participated. However, specialists such as Microbiologists (42.3%), Geriatric Medicine Specialists (73.0%) and Rehabilitation Medicine specialist (67.2%) had exceptionally low participation.

### Tumour board availability in public versus private hospitals

There was a statistically significant association between the type of hospital and the presence of Tumour Boards (**χ^2^ = 5.1727,** *p* = 0.023). Tumour boards were more frequently available in private hospitals (86.6%) compared to government hospitals (72.5%).

### Qualitative analysis on solutions to improve tumour board operations

**Figure 2.**
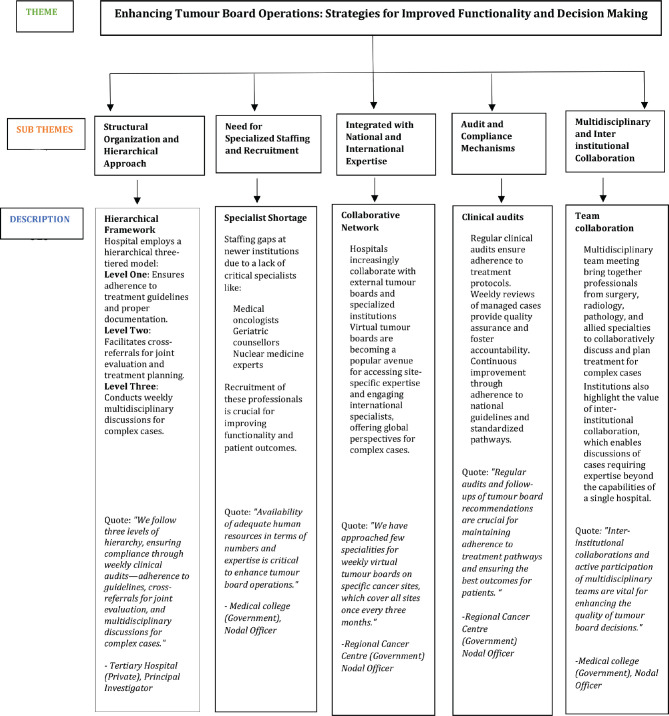
Qualitative analysis on solutions to improve tumour board operations.

The qualitative analysis of responses identified five key themes that illustrate the organisation, challenges and evolving practices of tumour boards in hospitals are presented below (Figure 2).

## Discussion

This study provides key insights into the characteristics, operational procedures and functioning of tumour boards within HBCR under NCRP. Evidence from this study highlights the significant disparities in tumour board practices across the institution with private hospital (86.6%) conducting tumour board meetings more frequently than public hospitals (72.5%).

The current study found that most of the institutes have single functional tumour boards (75.1%) and majorly weekly meetings (47.4%) were conducted. A study conducted in Odisha found that the presence of tumour board is needed for improvements in the management of advanced-stage disease and it has increased treatment compliance (>50%) [6].

The HBCR 2021 report highlights that advanced cancer stages are commonly diagnosed in India, especially for respiratory and digestive system malignancies with a significant proportion of case presenting with distant metastasis (e.g., lung cancer: 49.2% in males and 55.5% in females) [7]. One of the studies reported that the effectiveness of MTB meetings is dependent on a range of factors such as structural and functional components as well as the expertise of participants [6]. Our findings revealed that despite the high burden of late stage cancer cases in India, there is limited involvement of key specialists such as palliative care specialist, geriatric specialists and rehabilitation medicine specialists compared to global standards [4]. One another study reported that 96% of rectal cancer patients discussed at the MTB meeting had complete preoperative staging compared to 63% of patients not discussed [8]. Although some studies have shown a significant association between MTB discussion and improved quality of care, a systematic review suggests that after controlling for medical factors there is limited evidence directly linking MTB meeting to increased survival [9]. These findings suggest that there is a need for greater integration of multidisciplinary care, especially for complex cancer cases, in order to provide comprehensive treatment [10].

In the current study, the majority of hospitals (51%) reported that they implement the recommendations of the tumour board most of the times. Another study found that 64% of all cases fully adhered to the tumour board’s treatment recommendations, while 21% showed deviations, primarily due to comorbidities and varying patient needs [11]. Evidence showed that adherent to recommendations given by the tumour board had better recurrence-free survival [12].

Our study also highlighted that there is an inconsistent follow up mechanism (51.8%) followed by the MTB institutions, which reduces the ability to assess the long term impact of the recommendations made during MTB meetings. This is due to that fact that cancer care is fragmented. Patient access two to three hospitals for each episode of cancer leading to high drop outs [13]. In addition to that, major cancer centres are more concentrated in urban areas with gap in infrastructure and human resources, which hinders the delivery of higher quality of care and also leading to loss to follow up [14]. Efforts to implement MTB decisions and follow up at the hospital level remain inadequate.

Many hospitals in our study still depend on physical records (63.5%) rather than electronic records (26.3%), which reduces the effectiveness of data management and tracking [4, 15]. Utilisation of electronic health records (EHR) are in transformation stage in India. Use of physical records by the physician is due to various reasons like high patient load, time insufficiency and non-user friendly interface of technology. In addition to that written case summaries are also being missed. Implementation of EHR will help to track the recommendations discussed in MTB meeting and also improves the treatment decision. However healthcare sector is striving hard to shift towards digital records and government has introduced initiatives like National Digital Health Mission to promote digital health and also the data reporting for cancer registry in developed countries operate through EHR and insurance database records making the data available in real time [16, 17].

Greater awareness is needed, particularly among hospital lacking traditional tumour board setups, to encourage participation in virtual tumour board discussions [18]. Underutilisation of virtual tumour boards and limited cross – hospital collaboration (only 5%) affect the decision making for complex cancer cases [19]. The National Cancer Grid has initiated virtual tumour boards and many hospitals have joined this initiative [20].

Most private hospitals are well equipped with multidisciplinary care facilities and cater to higher socio economic class, which could explain the higher frequency of tumour board meeting (86.6%) in these institutions. A study analysing palliative care services in India found out that merely 26% of participating centres were in the government sectors indicating that non – government and private sectors are responsible for most of the palliative care services in the country [21]. These findings highlight the disparities in tumour board practices due to resource constraints, administrative priorities and staff availability in public hospitals.

Qualitative findings of the study revealed that a hierarchical approach could be adopted by hospitals to implement tumour board discussions at their own centres. Establishing such a system could ensure sustainability, while parallel audit mechanism may be used to assess improvement in clinical care. Adequate infrastructure, logistics and co – ordination between various specialities could largely facilitates the discussions. Additionally, hospitals with lesser amount of patients could participate in cross – hospital collaboration with other institutions to optimise resource utilisation and expertise sharing. Another study on exploring perspectives of physicians in tumour boards beyond clinical decision found that the multidisciplinary discussions provide opportunities for education, research and by building co-operation between colleagues promotes collective professional fulfilment [22].

## Strengths and limitations

This study provided important insights into the tumour board practices across various hospitals, identifying both strengths and areas that need improvement. It also provides a comprehensive overview of the current state of tumour board operations across India.

### Limitation

The questionnaire was reviewed for content validity by a panel of subject-matter experts; however, no additional psychometric analyses were conducted. This study used self-reported data from the hospital staff, which could have reporting/information bias. In addition to this, the patient outcomes are not linked to tumour board evaluation leading to limited interpretation on the adherence and utility of tumour board recommendations made during discussions. Also, the short duration of data collection (1 month) may limit the representativeness of ongoing MTB practices.

## Conclusion

The study revealed that 79.7% of hospitals had active tumour boards, with 52.5% maintaining a designated secretariat for operational support. Most tumour board (73.7%) held in-person meetings, but cross – hospital case discussion was rare (5.1%). Documentations were primarily physical (63.5%) and only 51.8% had a follow up system for treatment recommendations. Tumour boards were more prevalent in private hospital (86.6%) than public ones (72.5%), which highlight the disparities in access. Strengthening multidisciplinary participation, standardising MTB protocols/meetings, adoption of EHR, follow-up mechanisms, cross-hospital collaborations/virtual tumour boards are recommended to improve tumour board effectiveness.

## Conflicts of interest

Nil.

## Funding

Nil.

## Presentation in meeting/conferences

Nil.

## Ethical approval

Institutional Ethical Committee approval was obtained from ICMR-NCDIR Institutional Ethics Committee (No.NCDIR/IEC/3081/2024), Date of IEC approval - 20 September 2024.

## Declaration statement

All authors have read and approved the manuscript for submission to publication. All authors have met the requirements for authorship and represents honest work.

## Author contributions

**Table:** 

	Contributor 1	Contributor 2	Contributor 3	Contributor 4
Concepts	✓	✓		✓
Design	✓	✓		✓
Definition of intellectual content	✓	✓		✓
Literature search	✓	✓		
Data acquisition	✓	✓	✓	✓
Statistical analysis	✓			
Manuscript preparation	✓			
Manuscript editing	✓			
Manuscript review	✓	✓	✓	✓
Guarantor	✓	✓	✓	✓

## Figures and Tables

**Table 1.** Profile of tumour boards.

Variable	Frequency (*n* = 137)	Percentage (%)
Number of functional tumour boards		
One	98	71.5
Multiple	39	28.5
Mode of conduct		
In-person	101	73.7
Virtual	2	1.5
Hybrid	34	24.8
External specialist involvement		
Sometimes as per need	48	35.0
No	89	65.0
Cross-hospital case discussion		
Always	7	5.1
Sometimes	49	35.8
Never	81	59.1
Designated secretariat		
Yes	72	52.5
No	65	47.5

**Table 2.** Operational procedures of tumour boards.

Variable	Frequency (*n* = 137)	Percentage (%)
Annual calendar of meetings		
Yes	87	63.5
No	50	36.5
Frequency of meetings		
Daily	4	2.9
Weekly	65	47.4
Bi-weekly	23	16.8
Thrice a week	2	1.5
Monthly	4	2.9
As per need basis	26	19.0
Others	13	9.5
Criteria for case presentation		
Special cancer cases	72	52.5
All cancer cases	65	47.5

**Table 3.** Functioning and outcome tracking of tumour boards meetings.

Variable	Frequency (*n* = 137)	Percentage (%)
Documentation method		
Electronic	16	11.7
Physical	87	63.5
Both	34	24.8
Recommendation communication		
Electronic medical record notes	23	16.8
Written summaries distributed to providers	52	38.0
Direct communication with referring physician	48	35.0
Others	14	10.2
Follow-up system on recommended treatments		
Yes	71	51.8
No	66	48.2
Implementation of tumour board recommendations		
Always	40	29.2
Most of the time	81	59.1
Sometimes	13	9.5
Occasionally	3	2.2
